# Mineral Biofortification of Vegetables as a Tool to Improve Human Diet

**DOI:** 10.3390/foods10020223

**Published:** 2021-01-21

**Authors:** Camila Vanessa Buturi, Rosario Paolo Mauro, Vincenzo Fogliano, Cherubino Leonardi, Francesco Giuffrida

**Affiliations:** 1 Dipartimento di Agricoltura, Alimentazione e Ambiente (Di3A), University of Catania, Via Valdisavoia, 5-95123 Catania, Italy; camila.buturi@phd.unict.it (C.V.B.); cherubino.leonardi@unict.it (C.L.); francesco.giuffrida@unict.it (F.G.); 2 Department of Agrotechnology and Food Sciences, Wageningen University & Research, P.O. Box 16, 6700 AA Wageningen, The Netherlands; vincenzo.fogliano@wur.nl

**Keywords:** fresh consumed vegetables, agronomic biofortification, mineral

## Abstract

Vegetables represent pillars of good nutrition since they provide important phytochemicals such as fiber, vitamins, antioxidants, as well as minerals. Biofortification proposes a promising strategy to increase the content of specific compounds. As minerals have important functionalities in the human metabolism, the possibility of enriching fresh consumed products, such as many vegetables, adopting specific agronomic approaches, has been considered. This review discusses the most recent findings on agronomic biofortification of vegetables, aimed at increasing in the edible portions the content of important minerals, such as calcium (Ca), magnesium (Mg), iodine (I), zinc (Zn), selenium (Se), iron (Fe), copper (Cu), and silicon (Si). The focus was on selenium and iodine biofortification thus far, while for the other mineral elements, aspects related to vegetable typology, genotypes, chemical form, and application protocols are far from being well defined. Even if agronomic fortification is considered an easy to apply technique, the approach is complex considering several interactions occurring at crop level, as well as the bioavailability of different minerals for the consumer. Considering the latter, only few studies examined in a broad approach both the definition of biofortification protocols and the quantification of bioavailable fraction of the element.

## 1. Introduction

Many nutritional recommendations for human well-being and disease prevention have highlighted dietary styles based on the growing consumption of fresh fruits and vegetables and the reduction of simple carbohydrates, sodium, and saturated and trans-fats consumption [1].

In order to maintain a good health, people require several mineral nutrients that must be included in the diet. The essentiality of minerals can be demonstrated by the fact that vitamins cannot be absorbed solely or work in the absence of specific minerals, which are necessary in many physicochemical processes [2].

Deficiencies of specific mineral elements affect, in both underdeveloped areas and industrialized countries, up to two-thirds of the world’s population [3,4,5] and the insufficient intake can cause severe damage to people’s health [6]. For instance, in Europe and Central Asia, malnutrition problems related to diets with low micronutrient contents are increasing the number of women and children with anemia. In fact, iron and iodine deficiency disorders are the most common forms of malnutrition [7]. Besides, a recent study conducted in South Italy showed that the population has low intake of calcium and potassium [8].

Food, mainly plant-based, is the source of all important minerals, therefore it is important to keep on a regular basis a good and balanced diet that can provide the adequate proportion of minerals [9]. The enrichment of food with health related compounds and mineral elements could, however, be considered a strategy to fight undernourishment or to face with specific nutritional need [10].

In the case of not processed food, such as vegetables, the only option to enhance the nutrient content of products in preharvest using improved genotypes or adopting specific agronomical techniques [11].

The increasing interest in the enrichment of fresh consumed vegetables with mineral elements has encouraged intensive research activity focusing on the elaboration of suitable application protocols. This review describes developments in agronomic biofortification of vegetables with reference to some mineral elements often lacking or not adequately present in human diets, i.e., calcium (Ca), magnesium (Mg), iodine (I), zinc (Zn), selenium (Se), iron (Fe), copper (Cu), and silicon (Si). After synthetically considering the role in human nutrition and in plant physiology, this review aims to discuss the most successful agronomic strategies to increase the amount of the considered minerals in the edible portion of vegetables.

## 2. The Role of Vegetables for Human Health and How Biofortification Can Have an Impact

Plant foods make up a substantial part of the human diet and they provide most of calories, nutrients, and bioactive compounds necessary to keep a healthy status and prevent diseases. Vegetables are one of the pillars of a plant-based good diet, providing in particular dietary fiber, phytochemicals (such as vitamins, antioxidants), and minerals [12,13]. Minerals are considered essential nutrients: they are not synthesized by humans and must be obtained from the diet. Humankind evolved thanks to the dietary assumption of a significant number of vegetables and their insufficient intake is one of the reasons for many noncommunicable diseases, which are spread in Westernized societies. As an example, potassium, calcium, selenium, and iodine obtained through a vegetable-rich diet, can contribute to maintaining good blood pressure, bone strength, hormonal production, heart, and mental health [14,15]. In a recent study carried out in the UK, a data analysis from more than 40,000 people showed that changes in fruit and vegetable consumption may not only benefit physical health in the long-run, but also mental well-being in short term [16,17], besides the general population these benefits were also observed in cancer survivors [18]. On the other hand, vegetables play an important role in the economy, fighting poverty, hunger, and undernutrition, since they can be locally cultivated and consumed in a high diversity of shapes, sizes, colors, and tastes [12,19,20].

Nonoptimal intake of micronutrients and undernutrition, the so called hidden hunger, can be particularly severe for people following restricted diet for religious, ethical, or medical reasons [4,5,21]. Health authorities have established dietary reference intakes (DRI) based on recommended daily allowances (RDA) and tolerable upper levels (UL). As general principle, strategies that address vitamin or mineral deficiency must aim to achieve the DRI for each component without exceeding the UL [22].

However, the actual contribution of phytochemicals and minerals to human diet is not only related to their concentration in a certain plant tissue. The micronutrients must be released from the food matrix during the passage in the gastrointestinal track, absorbed into the blood and transported to their target tissues [23]. In fact, only the fraction released from the plant tissue become eventually available for absorption. This fraction is indicated as bioaccessible and to increase the bioaccessibility of plant phytochemicals and minerals is a promising target of agronomical strategies to improve the nutritional quality of vegetables [24].

Vegetable consumption should increase in the coming years for sustainability and health reasons. To deal with the rise of global population, more sustainable food sources will be needed [25]. According to Schreinemachers et al. [15], the most important vegetables in the current global economy are tomatoes, cucurbits (pumpkins, squashes, cucumbers, and gherkins), alliums (onions, shallots, and garlic), chilies, spinach, potatoes, carrots, and brassicas, therefore, it makes sense to focus the biofortification efforts on these species.

## 3. Biofortification of Vegetables

The approaches to address micronutrient malnutrition are different; medical supplementation and product fortification are the most commonly adopted. Fortification is the process of food enrichment with nutrients, adopting different methods during processing. However, in some contexts, fortification is challenged due to poor investments, infrastructure, and delivery system [26]. Under these conditions, an alternative strategy is to adopt new genotypes, characterized by improved compositional profiles, or to tailor specific agronomic techniques aimed to enhance the content of specific health effective compounds in widespread crops [26]. While this can be considered an option for products which are transformed before they are used (e.g., staple foods), for fresh consumed products, such as vegetables, biofortification is the only choice to improve the content of health-related compounds in the edible portion.

Among the different strategies to obtain biofortified vegetables there are agronomic and genetic approaches, the latter can be done either through conventional breeding or transgenic methods [27,28]. The objectives are to increase in the edible portion the minerals content or other specific health related compounds. Transgenic programs involve biotechnology studies that allow to genetically modify a species, to obtain a plant with targeted characteristics (i.e., higher content of specific nutrients). Even though this approach could be cost effective in the long run, it is the least employed methodology today because the phase of research and development is still very slow and expensive. In any case, in developed countries the higher prices involved in the production of biofortified vegetables is countered by the achievement of a premium product with a superior nutritional quality, that can satisfy the new consumers’ demand willing to pay for a healthier way of eating [29]. In addition, some countries have restrictive laws, that forbid genetically modified organisms (GMOs). Along the same lines, there is the option to cross different genotypes, with the aim to introduce in new cultivars desirable traits naturally occurring in plants. This genetic approach (traditional breeding) has been performed for decades and can allow to create new varieties with a higher content of certain nutrients. In this case, the limitation is to find the desired characteristics in the available genetic resources [30]. On the other hand, breeding programs, even when effective, may eliminate their effect due to the high renewal rate of cultivars coming from the large number of new introductions made by the vegetable seed industry [31].

Biofortification programs carried out through the agronomic approach are the best option, since they involve simple techniques to accumulate or to stimulate the production of specific compounds at plant level. A substantial part of the biofortification research that has been carried out in the last decades focused the attention on specific compounds such as vitamins and amino acids, rather than minerals [4,13,32,33,34]. A variety of biofortified products with vitamins or their precursor include banana, mango, sweet potato, wheat, and cauliflower [35]. In the same line, biofortification with amino acids proved to be effective in producing high lysine-rice, using the double strategy of maximizing its biosynthesis and minimizing its catabolism [36]. Besides, evidence shows that sulfur fertilization on wheat, barley, and potato can increase the sulfur-containing amino acids (SAAs) methionine and cysteine content in its edible part. In the same way, the application of nitrogen plus potassium has potential in increasing carotenoid content in carrots [37].

However, besides the increase of the content of some specific compounds (e.g., antioxidants) with controlled doses of stressors [38], agronomic biofortification consists in increasing or optimizing the application of mineral elements to the crop in order to increase the corresponding content in the edible organs. In this case the focus is on setting up the form of the mineral, the concentration, and the application form; indeed, certain mineral forms or quantities can cause indirect effects, damaging or compromising a crop [5,27].

## 4. Agronomic Mineral Biofortification

The production of vegetables is carried out in very diversified agronomic contexts as regards crop cycles, soil conditions, or growth environments. Agronomic approaches to increase the concentration of minerals in edible organs generally rely on the supply of mineral fertilizers and/or improvement of the mobilization and solubilization of mineral elements in the rhizosphere [27]. Vegetable crops are generally grown in agro-systems characterized by a high degree of intensification of the production processes and in which the supply of nutrients is increasingly based on the use of fertigation, soilless cultivation, and foliar fertilization. These alternatives offer different opportunities to implement targeted biofortification programs. In the case of the application of mineral elements by fertigation on soil cultivated crops, some interference may derive from element availability for the plant (phytoavailability), therefore selecting mineral forms and concentrations may have a relevant importance [27,32]. One alternative strategy to overcome the low mineral phytoavailability into the soil is the cultivation through hydroponic systems (soilless cultivation). The possibility of optimizing limited water resources and cultivating in the absence of suitable agricultural soils, has led to a considerable spread of soilless cultivation systems. It has been observed, for example, that hydroponic cultures can be among the best options to increase the nutrient content of plant tissues [39,40]. In the case of minerals not readily translocated to the edible tissues, such as for crops grown on soil and/or for minerals with scarce mobility, another alternative is the use of foliar fertilization [41].

### 4.1. Calcium

In human health, calcium (Ca) is required in several systems, like musculoskeletal, nervous and cardiac. It is essential to maintain good bones, teeth, and mineral homeostasis. It also acts as a cofactor in many enzyme reactions and contributes to the function of the parathyroid gland [42]. The RDA of Ca ranges between 1000 and 1300 mg day^−1^. The UL for adults is 2500 mg day^−1^ [43]. Calcium is an important nutrient for plant metabolism, involved in structural functions of cell, acting as a counter-cation for organic and inorganic anions trafficking across the tonoplast and as an intracellular, cytosolic messenger [44]. It is one of most abundant nutrients in the earth’s crust, with an average concentration of about 36.4 g kg^−1^[45]. Ca^2+^ concentration in the soil solution is usually enough (0.1–20 mM) to meet the plants’ demands or, in neutral and calcareous soils, to exceed their requirement, thus leading to Ca accumulation in the vicinity or inside the roots [44]. However, some Ca-deficient conditions can sometimes be encountered, especially in highly weathered tropical soils or in saline/sodic soils. Calcium is absorbed as divalent cation by the root apex and/or regions of lateral shoot initiation [46], where Casparian band between endodermal cells is absent or disrupted, and/or the endodermal cells surrounding the stele are not suberized [47]. Once inside the plant, Ca moves primarily through the xylem [46] with the water flow driven by transpiration [48,49], either as Ca^2+^ or complexed with organic acids [50]. However, Ca^2+^ movement inside the xylem vessels cannot be explained simply in terms of mass flow, as Ca^2+^ ions are also absorbed by adjacent cells and are complexed to nondiffusible anions in the xylem walls [48]. Due to its slow phloematic mobility, this element is present at lower concentrations in mostly phloem-fed organs (e.g., young leaves, fruits, and tubers) than in the older leaves (≈10-times less). Considering the mineral partitioning inside the plant, leafy vegetables can play a primary role in the dietary intake of Ca, so being possible targets for Ca biofortification [51]. This last point should be addressed at increasing the Ca content of the edible portions, without adversely impacting both plant growth and production costs [27]. Most plant species can accumulate high Ca contents in leaf laminae (up to 100 g kg^−1^ DW) without any symptoms of toxicity, because Ca exceeding plant’s needs is detoxified by sequestering as insoluble Ca oxalate and deposited either in the cell wall or stored inside the vacuole [44,47]. Depending on the plant species, tissues, and growing conditions, Ca concentration in plants varies between 1 and >50 mg kg^−1^. However, some species may have insufficient detoxification mechanisms, so their growth can be severely depressed at high Ca tissue content [44]. Excessive Ca can cause toxicity symptoms such as the presence of yellow flecks formed by crystals of calcium oxalate and growth inhibition, the latter can be observed even in calcicole species (plants occurring in calcareous soils) when submitted to a soil solution with a concentration higher than 10 mM Ca [46]. Strategies for Ca biofortification should include (i) increasing Ca supply to cells; (ii) increasing Ca uptake into cells; (iii) removing compounds making Ca unavailable and/or (iv) increasing Ca storage at the cellular and/or tissue level [27,52,53]. The application of Ca fertilizers can increase its concentration mostly in leafy vegetables (Table 1), whereas for grain, seeds, and fruits, sound indications are still to be reached. In 21-day old *Brassica rapa* plants grown on soil, the increased Ca supply to roots (compost mix supplemented with 0.4 vs. 3.5 g CaCl_2_ L^−1^) significantly enhanced its concentration in leaves (0.75 and 25 g kg^−1^ DW, respectively). The result was not influenced by the different supply of Mg fertilizer [54]. To reduce the effects of different soil characteristics (e.g., minerals concentration, pH) on Ca availability, soilless cultivation on inert substrates or water (e.g., floating system) allows a better control of the ion concentration in the root environment. In some leafy vegetables, D’Imperio et al. [24] increased the Ca concentration by adding calcium phosphate and calcium chloride in the nutrient solution (from 100 to 200 mg L^−1^), determining an increase of Ca concentration in leaves of basil (≈15%) and mizuna (≈12%), but not in tatsoi or endive (Table 2). Moreover, the biofortification process did not influence their oxalate content nor Ca bioaccessibility. A higher Ca content (up to 5-fold higher than control) in lettuce (*Lactuca sativa* L.) grown in a floating system was obtained by Borghesi et al. [55] with a nutrient solution containing 800 mg Ca L^−1^ (as CaCl_2_), compared to the control with no Ca addition. However, the high salt content increased both the Cl concentration and electrical conductivity of the nutrient solution, so reducing the marketable quality and yield (−32%). Foliar applications of soluble Ca fertilizers are commonly made for several horticultural crops, to prevent Ca-deficiency disorders. However, only few experiments refer to Ca biofortification through foliar applications. Moreover, these applications are expected to have limited effects on Ca content of roots, tubers, and seeds, because of the typical translocation patterns of the element. In one of few experiments, Yuan et al. [56] observed a significant increase of Ca concentration in lettuce sprayed three times every 20 days with 120 mg L^−1^ of CaCl_2_ compared to 60 and 180 mg L^−1^ (21.4% and 5.2%, respectively), although this effect was genotype-dependent. Overall, Ca biofortification of vegetables using Ca chloride proved to be effective in the majority of the studied leafy crops even if negative effects on yield cannot be excluded; besides, one of the main challenges is related to the presence of oxalate, which can partially limit Ca bioavailability.

### 4.2. Magnesium

In human health, magnesium (Mg) is essential in maintaining muscle tone and blood pressure. It participates in glycemic control, neuromuscular function, and myocardial contraction. It is also involved in the energy metabolism, besides being a cofactor of many enzymes [57]. The RDA for Mg ranges between 320 and 420 mg day^−1^. The UL for adults is 420 mg day^−1^ [43]. Magnesium is a divalent cation and it is essential in plants because of its ability to interact with strongly nucleophilic ligands, it participates in the processes of enzyme regulation, pH cellular, and cation–anion balance, besides being a key metal in chlorophyll structure [58]. Magnesium is relatively mobile in soils, where its average concentration can vary between 0.5 and 40 g kg^−1^ [59], with a worldwide average of 5 g kg^−1^. In addition to passive diffusion, as it happens with others divalent cations, Mg is actively absorbed by roots through permeable cation channels [60]. Regarding leaf uptake, younger leaves are more likely to absorb Mg than the aged ones [61]. Mg^2+^ transporters in higher plants are thought to be derived from the CorA transport system, acting as a gate locking it when the Mg concentration in the cytosol is increasing or opening otherwise [44,62]. Concentration of Mg in the metabolic pool of leaves is supposed to be between 2 and 10 mM, while free Mg concentration is expected to be lower (around 0.4 mM). For an optimal growth, plants demand between 1.5 and 3.5 g kg^−1^ of Mg in vegetative fractions [44]. Even though toxicity with Mg is rare, concentrations above 20 mM proved to be phytotoxic, causing symptoms like coppery colored leaves, decrease in starch contents, and growth reduction [63]. In contrast to the translocation difficulties observed for Ca, Mg shows a high phloem mobility and the application of Mg fertilizer can efficiently increase its concentration in leaves, tubers, fruits, seeds, and grains [27,44], making Mg agronomic biofortification of vegetables a feasible option to fight cases of malnutrition. As indicated in Table 1, plants of Indian colza (*Brassica rapa* ssp. *trilocularis*) submitted to different Mg biofortification protocols, showed on average a 3.6-fold increase in Mg content of leaves, when compared to untreated plants. In one experiment, after growing Indian colza plants on peat with a low (0.20 g L^−1^) or high (3.04 g L^−1^) Mg chloride (MgCl_2_) concentration, leaf content increased up to 12 mg Mg kg^−1^ DW. However, the increase was 50% lower when plants received simultaneously a high dose of CaCl_2_ (3.04 g), showing a possible negative interaction between Mg and Ca [54]. Similarly, Blasco et al. [64] submitted *Brassica rapa* plants to different nutrient solutions. When comparing the application of a low (4.86 mg L^−1^) and a high (486.1 mg L^−1^) dose of Mg (as MgCl_2_) in the nutrient solution they noticed a 12-fold increase in the Mg content of shoots, passing from low to high dose. The same authors tested the interaction with other minerals and concluded that the Mg concentration in shoots increased with high Zn (500 µM) and low Ca (0.4 mM) supplies and decreased at high Ca (40 mM) supply. Another biofortification study of Mg was conducted applying doses of 0, 50, 100, 150, and 200 mg Mg dm^–3^ soil (as magnesium sulfate, MgSO_4_·7H_2_O) on growth of onion plants (*Allium cepa* L.) [65]. The maximum Mg content in bulbs was obtained at the dose 150 mg dm^–3^, i.e., almost 2-times higher than the untreated plants. However, this dose negatively affected the crop yield, and also caused a reduction in the uptake of Ca and potassium (K), showing that the antagonism between these minerals should be carefully evaluated. Therefore, the authors suggest using the Mg-100 dose, as it allowed to increase the Mg content of the bulbs (up to 1.4-fold, when compared to control), with a contextual increase in crop yield (up to 38%). There is evidence that fertilization of Mg via foliar spray can act to improve crop yield and quality [66,67]. The few studies on Mg biofortification show that both MgSO_4_·7H_2_O and MgCl_2_ are effective in enhancing the element content in vegetables. However, Mg biofortification should be carefully managed considering its interaction with Ca, since high Ca content can inhibit Mg uptake by plants.

### 4.3. Iodine

Iodine (I) is essential for humans; it is required in the synthesis of the hormones thyroxine and triiodothyronine that are produced in the thyroid gland and are responsible for regulating growth and development, besides maintaining the basal metabolic rate [68]. The RDA of I is 150 µg day^−1^, whereas the UL for adults is 1100 μg day^−1^ [69]. Typical I concentration in soils is between 0.5 and 20 mg kg^−1^, and even though not essential to plant growth, it can be absorbed and translocated within the plant tissues. Plant leaves absorb I through stomata (60%) and leaf surface (40%), but I losses can occur too, due precipitation, wind, and tissue decay; the remaining can be partially transported via phloem to the other plant organs, including roots [70]. According to Smolèn et al. [71], leaves absorption occurs due the organophilic nature of I and its interaction with cuticular waxes or oxidation of I- (iodide) to I_2_ (iodine), facilitating I penetration into the cuticle. It is known that root absorbs iodide better than elemental I or iodate, especially in plants grown in hydroponic systems. This I is majorly retained into the roots, but when in nutrient solution with concentrations higher than 0.01–10 µM it can also be translocated to the shoots [70,72]. In fact, I is efficiently transported into the xylem, transport in plants is analogous to chloride movement, I^–^ uptake being catalyzed by H+/anion symporters and released into the xylem by anion channels [27]. Concentration of I in plants can be zero or extremely low, about 30–100 µg kg^−1^ FW [73]. Depending on plant species, a nutrient solution with concentrations higher than 10–100 µM can already be phytotoxic and inhibit plant growth [70]. In general, the different I chemical forms present the following phytotoxicity order: (I_2_) > (I^−^) > iodate (IO_3_^−^) [74]. Horticultural crops are the best candidates for I biofortification, because of their ability to absorb and accumulate exogenous I into the edible fractions [75]. As reported in Table 1, once submitted to different biofortification protocols, leaf species such as basil and Chinese cabbage, showed an average I increase higher than 100-fold in their edible tissues, while cabbage, lettuce, mizuna, mustard, and spinach resulted in increases varying from 5 to 91 times. Average accumulation of I in vegetable fruit species was higher than 100-fold in both tomato and cowpea. Tuber species such as potato, showed a 13-fold average increased in I content, while root vegetables such as carrot presented a much higher average increase (greater than 100-fold). Biofortification of I through repeated foliar spray has been successfully performed in carrot and mustard plants [76,77]. Higher efficacy of lettuce iodine biofortification was noted Smolèn et al. [71] after foliar application, rather than adding the element to the nutrient solution. On the contrary, Caffagni et al. [75] demonstrated that, even though it is possible to enhance the I content of tomato fruits through KIO_3_ foliar spray, better results were observed through fertigation with a 5 mM solution of KI; this allowed to achieve a 249-fold I increase in this vegetable. When grown in water culture, lettuce plants grown with 90 μg I L^−1^ as potassium iodide (KI) showed better biofortification results than plants submitted to the same amount of I as potassium iodate (KIO_3_), with the result consisting of 30-times more I in leaves than untreated plants [78]. Low doses of I, such as 0.25 mg L^−1^ (KI) or 0.50 mg L^−1^ (KIO_3_) in the nutrient solution are enough to achieve around 7 mg kg^−1^ DW of I in strawberry fruits, compared to 0 in the control, improving plant growth too [79]. Analogous results were observed in several leafy vegetables (e.g., broccoli raab, curly kale, mizuna or red mustard) when submitted to low doses of iodine (0.75 mg L^−1^, 5.9 µM KIO_3_) through fertigation, showing an increase ranging from 390 to 471 µg kg^−1^ FW [80]. However, high I levels (50 mg L^−1^) in the nutrient solution, proved to increase the I content in carrot up to toxic amounts for humans (9 mg kg^−1^ FW) showing also phytotoxic effects on plants [76]. In addition, I biofortification should be carefully evaluated, since there is evidence that I can decrease Cu uptake by plants [73]. However, even though insufficient phloem loading and high volatilization rates could limit I accumulation, both K iodate and K iodide have successfully increased the I content in horticultural products.

### 4.4. Zinc

In human health, zinc (Zn) is essential for maintaining the structure and activity of many enzymes, besides playing a key role in the synthesis of nucleic acids and proteins. It acts in cell differentiation, glucose use, and insulin secretion [81]. The RDA of Zn ranges between 9 and 14 mg day^−1^, whereas the UL for adults is 40 mg day^−1^ [69]. Zinc is essential in plant metabolism, as it plays a key role in chloroplast development and function through the Zn-dependent activity of SPP peptidase and repair of photosystem (PS I) I, besides participating in enzyme activation process such as RNA polymerases and superoxide dismutase, protein synthesis and metabolism of carbohydrate, lipid, and nucleic acid [82]. Although most of the world’s cultivated soils contain enough Zn to sustain its accumulation in plants’ edible portions (between 10 and 100 mg kg^−1^), Zn phytoavailability is a factor often limiting its uptake by roots, so that it has been estimated that about one-fifth of the world’s population actually suffers from Zn deficiency [83]. Under these conditions, agronomic strategies are aimed to improve the Zn phytoavailability into the soil, e.g., by correcting soil alkalinity, implementing more proper crop rotations, introducing beneficial soil microorganisms, or delivering phytoavailable Zn through the application of Zn-fertilizers to soil or foliage [83]. Zinc is absorbed by the plants from the soil solution primarily as Zn^2+^ (Strategy I plants) or complexed with organic ligands released by roots (phytometallophores), a mechanism which is restricted to cereals (Strategy II plants) [84]. Once inside the plant, xylem loading occurs either via symplast and apoplast, whereas in the xylem sap Zn is transported in its ionic form or in form of metal complexes with asparagine, histidine, organic acids, and nicotianamine [85]. Similarly, phloem Zn redistribution to various organs is thought to be affected either as divalent cation or in complexed forms with nicotianamine, malate, or histidine [27]. Due to its low phloematic mobility, Zn-supplied plants through the rhizosphere show a decreasing Zn concentration in the order shoot ≈ root > fruit, seed, tuber, thus showing a penalty on phloem-fed organs [86]. For this reason, root crops and leafy vegetables are thought to have the greater potential to increase dietary Zn uptake [83]. It must be pointed out that despite the low Zn phloematic mobility, Zn translocation through phloem for several plant species after application to foliage has been found to be nutritionally considerable for their growth and development, especially when cultivation occurs on substrates with low Zn phytoavailability [87]. Plants markedly differ in their ability to accumulate Zn in their tissues, but as a general rule, most crops require a leaf Zn concentration higher than 0.015–0.030 g kg^−1^ DW to reach their maximal yield. However, phytotoxicity symptoms are usually noticed at concentrations greater than 0.1–0.7 g kg^−1^ DM, depending on the species and exposure time [83]. When toxicity levels are attained, plants show an array of heavy metal stress responses such as growth and yield inhibition, leaf chlorosis and necrosis, restricted stomatal conductance and CO_2_ fixation, changes in chlorophyll structure and concentration [88], so the higher threshold concentration actually represents a physiological limit to the biofortification achievements. Nonetheless Zn hyperaccumulation capacity has been observed in members of Brassicaceae, Caryophyllaceae, Polygonaceae, and Dichapetalaceae, whereas a greater Zn susceptibility has been noticed in the Linaceae, Poaceae, and Solanaceae [84]. Common inorganic Zn-fertilizers include ZnSO_4_, ZnO, and synthetic chelates [27] such as Zn-EDTA, Zn-DTPA, or Zn-HEEDTA. When foliar applications are concerned, the Zn compounds used must be highly soluble and enter rapidly into the leaf apoplast, in order to promote Zn translocation to phloem-fed organs, so avoiding possible interferences with mesophyll metabolism [86]. Due to their ability to hyperaccumulate Zn, leafy *Brassicas* have been extensively studied in biofortification protocols (Table 1). In kale leaves (*Brassica oleracea* L. var. *acephala*), de Sousa Lima et al. [89] reported up to a 28-fold increase of Zn concentration by providing the crop with 300 mg Zn kg^−1^ soil. After applying 22.7 kg ha^−1^ of Zn (in the form of Zn sulphate, ZnSO_4_·7H_2_O) to the soil, Mao et al. [90] observed a significant increase in the Zn concentration of the edible portions of canola (*Brassica napus* L.) and cabbage (*Brassica rapa* L. Chinensis Group) (by 25% and 200%, respectively). Zinc biofortification through foliar spray has been successfully performed in arugula (*Eruca sativa* L.) using 1.5 kg ha^−1^ of ZnSO_4_·7H_2_O, with a resulting +94% increase of leaf Zn concentration [91]. Among non-Brassicas leafy vegetables, in a study conducted by Barrameda-Medina et al. [92] hydroponically cultured plants of lettuce (*Lactuca sativa* L.) supplemented with 100 µM ZnSO_4_·7H_2_O in the nutrient solution showed a 251% increase in leaf Zn concentration. Simultaneously biofortification programs must take into account that high Zn concentration on soil cultivated crops can negatively affect Fe absorption and improve the content of Mn and of amino acids [89]. In conclusion, Zn biofortification, especially in the form of sulphate is promising in increasing the mineral content in vegetable products.

### 4.5. Selenium

Selenium (Se) is an essential trace mineral, constituent of the selenoproteins responsible for important enzymatic functions. The function of selenoproteins in the human metabolism is most commonly connected to immunocompetence and cancer prevention, but it is known that Se functions go above that, as this mineral plays an important role in fertility and reproduction, brain functions, mood, thyroid health, and cardiovascular diseases [93]. The RDA of Se is 55 µg day^−1^, and the UL for adults is set at 400 µg day^−1^ [69]. Selenium is not considered a micronutrient, but its appropriated use in plant nutrition can increase growth, stimulate seed germination, and contribute to protect several crops against pathogens and pests [94]. Soil concentration of Se is relatively low and it varies according to the type of rocks, being generally between 0.01 and 7 mg kg^−1^, with a worldwide average of 0.4 mg kg^−1^ [95]. Plants take up Se inorganically both as selenite (SeO_3_^2−^) and selenate (SeO_4_^2−^) [96]. Plant absorption and transportation of Se are active processes [97]. Into the roots, due to its chemical similarity to sulfur (S), selenate moves through high-affinity sulphate transporters, while selenite movement is partially mediated by phosphate transporters [97,98]. Translocation of Se from root to the aerial parts of the plant is more likely to happen as selenate, since selenite is more prone to be accumulated in roots. Leaf surface can absorb volatile forms of Se from the atmosphere [99]. Foliar application of Se at late growth stages seems to optimize the uptake, translocation, and distribution of Se into the edible portions of plants, whereas selenate is more efficiently accumulated in plant tissues than selenite [100]. The tolerable Se content in most plant species is between 10 and 100 mg kg^−1^ DW [101] and phytotoxic effects due to Se excess can compromise plant growth through damages to photosynthetic apparatus, photosynthesis inhibition, and over-production of starch [102]. In addition, secondary accumulators, also called Se-indicator, as some vegetables of the Asteraceae, Brassicaceae, and Fabaceae family, when supplied with exogenous Se can accumulate up to 1 g kg^−1^ DW, being a good target for Se biofortification [101]. Skrypnik et al. [103] reported that Se biofortification of basil through foliar application of sodium selenite (Na_2_SeO_3_) at 10 µM (4 applications starting from the 7º day after transplanting) enhanced the Se concentration in leaves to up 10.74 mg kg^−1^ DW (more than 700-times higher than untreated plants). Moreover, five applications of Na_2_SeO_4_ (0.633 mM), as foliar spray, from the six-leaf phase, resulted in lettuce leaves enriched with up to 40 mg Se kg^−1^ DW, around 40 times greater than the control [71]. In another study, radish plants sprayed with 5 mg Se per plant 7 days before harvest, as sodium selenate, were able to produce roots with 346.5 mg kg^−1^ DW of Se, meaning that the consumption of 1–10 radishes is enough to fulfill the daily human requirement [104]. Meanwhile, da Silva et al. [105] found that fertigation of radish plants could be more efficient than foliar spray, after treating plants with a low dose of Na_2_SeO_4_ (3.6 mg of Se per pot). They obtained roots with approximately 50 mg Se kg^−1^ DW, while the leaf spray of the same chemical (0.36 mg of Se per pot, 93 mL per pot) resulted in plants with approximately 15 mg Se kg^−1^ DW. Lettuce appears to be a good candidate for Se biofortification, as demonstrated by do Nascimento da Silva et al. [106]. In this experiment, plants submitted to fertigation at 25 µM Se L^−1^ (as sodium selenate) resulted in lettuce leaves with as much as 39.4 mg Se kg^−1^ DW, around 40 times greater than the control. While, higher application rates of both sodium selenate (Na_2_SeO_4_) and selenite (Na_2_SeO_3_) reached numbers that exceeded the RDA of Se. Similarly, tomato plants fertigated with 5 mg L^−1^ of Se as sodium selenate, were enough to obtain a significant increase in Se concentration of fruits (35.8 mg kg^−1^ DW), twice the concentration in the untreated plants. At the same time this dose allowed to achieve good physiological responses on plants, such as increased enzyme activity of catalase, glutathione peroxidase, and superoxide dismutase in fruits [107]. Selenium biofortification was successfully implemented in many vegetable crops, using Na selenate or Na selenite. Besides, possible antioxidant and antisenescence effects of Se can improve shelf-life during postharvest storage [108]. However, because of the high toxicity of Se, especially in the form of selenate, attention must be made regarding agricultural workers and product safety.

### 4.6. Iron

In human health, the main function of iron (Fe) is related to the synthesis of hemoglobin and myoglobin besides being essential to many metabolic processes such as oxygen transport, deoxyribonucleic acid (DNA) synthesis, and electron transport, it is also required for energy production [109]. The RDA of Fe ranges between 8 and 18 mg day^−1^, whereas the UL for adults is 45 mg day^−1^ [69]. Iron is a versatile, essential element in plant metabolism, whose biological functions are primarily based on the reversible redox reaction of Fe^2+^ (ferrous) and Fe^3+^ (ferric) ions, the ability to form octahedral complexes with various ligands and to change its redox potential in response to different environmental conditions. Due to this, Fe is involved in the transfer reactions at the base of life, since electron transfer chains of photosynthesis and respiration rely on iron–sulfur (S) clusters of the 2Fe–2S or 4Fe–4S type [110]. The concentration of this element in soil often exceeds plant requirements, being present at 20–40 mg kg^−1^ [111], but usually only a small amount of this is available for plant nutrition. Particularly in alkaline and calcareous soils, once applied through fertilization, Fe quickly becomes unavailable to roots absorption, because of precipitation, adsorption, and oxidation phenomena [112,113]. Plants have evolved two different strategies to acquire Fe from the growth substrate, based either on its reduction (Strategy I plants) or chelation with organic ligands (Strategy II plants) [114]. In nongraminaceous species (Strategy I plants), such as most of vegetable crops, organic acids and phenolic compounds released by roots chelate ferric Fe on the root surface (Fe^3+^), which is subsequently reduced to its ferrous form (Fe^2+^) to transport the element across the plasmalemma of root epidermal cells [27]. The Fe transportation within the plant occurs in chelated forms, mainly with citrate and malate in the xylem, and nicotianamine and its derivatives in the phloem [115]. This condition derives from the peculiarities of this metal, characterized by low solubility and high reactivity, so its transport inside the plant must be associated to proper chelating molecules controlling its redox states between ferrous and ferric forms [116]. The status of Fe into a plant is expressed by its quantity, redox state, speciation with chelating molecules, and its compartmentalization [117]. Chloroplasts represent the main pool of Fe within the cell, as they gather approximately 80–90% of cellular Fe [44]. This flows from the high Fe demand of the photosynthetic apparatus, and Fe-deficiency hampers the electron transfer between PSI and PSII, resulting in photooxidative damages [116]. Even though the range of Fe in leaves is between 50 and 150 mg kg^−1^ DW, Fe requirement is highly variable among species. For example, C4 species are more likely to require higher Fe amounts than C3 species; fast growing meristematic and expanding tissues need more Fe. On the other hand, Fe toxicity is reported in concentrations above 500 mg kg^−1^ DW, which can cause damages associated with formation of ROS, inducing the activity of antioxidative enzymes such as ascorbate peroxidase, besides damages to membrane and irreversible impairment of cellular structure, DNA, and proteins [44]. To improve Fe uptake agronomical solutions to make Fe available are acidification of soil [112] and/or use as Fe(III)-chelates synthetic fertilizers. Since the latter are expensive, their use is mainly restricted to soilless crops and to high added-value cash crops [117]. However, in the case of vegetable crops, the knowledge concerning Fe enrichment, and specifically biofortification, is still poor. One alternative to provide Fe to plants is the foliar spray even if, both adopting the chelated or the sulfate-salt form, a large fixation by cuticle can be observed [118]. Foliar spray of Fe sulphate heptahydrate (FeSO_4_·7H_2_O) proved to be effective to increase Fe content both in leaves and sink organs of herbaceous crops [119,120]. In tomato, leaf spray with a 9 mM FeSO_4_ solution increased by 3.8 times the Fe content in roots, mediated via phloem transport [121]. In a study conducted on potato, Kromann et al. [122] did not observe a positive relationship between Fe foliar spray with EDTA-chelated Fe and its concentration in tubers, thus the authors hypothesized that the limited effect was related to the Fe form used. As shown in Table 1, biofortification of vegetables with Fe through fertilization has been tested in few species. The use of EDDHA-chelated Fe up to 2.0 mM (112 mg L^−1^) proved to be effective in soilless cultivation of lettuce in increasing the Fe content of the leaves from 2.31 mg kg^−1^ FW (control) to 4.30 mg kg^−1^ FW [123]. In addition, it has been reported that low doses of Fe can enhance the accumulation of secondary metabolites such as chlorogenic acid, β-carotene, violaxanthin, or neoxanthin, thus leading to improved functional profiles of vegetables [123]. However, the authors observed a yield reduction of about 25%, which increased proportionally with the amount of Fe added to the nutrient solution. Overall, Fe biofortification has not been investigated enough to draw a clear picture. Using sulphate or chelate forms only in some cases enhanced mineral content in the edible part of vegetables, however, the increase was coupled with a yield reduction. Concluding significant insolubilization in the soil, limited translocation into the plant and accumulation into edible organs and negative effects on yield are the main constraints in Fe biofortification.

### 4.7. Copper

In human health, copper (Cu) importance is related primarily to enzymes function, contributing also to maintain cardiovascular integrity, lung elasticity, normal development of connective tissue and nerve coverings, neovascularization; it has also neuroendocrine and immune functions and it is involved in the Fe metabolism too [124]. The RDA of Cu ranges between 1.0 and 1.6 mg day^−1^, while the UL for adults is 10 mg day^−1^ [69]. Copper is a redox-active transition metal that under physiological conditions exists as Cu^2+^ and Cu^+^ [125]. In plants, it is essential to many physiological processes like photosynthesis, respiration, C and N metabolism, and protection against oxidative stress. It acts as cofactor of numerous proteins and in plants it is mainly present in complexed forms, the concentration of free Cu^2+^ and Cu^+^ in the cytoplasm being minimal [44]. The worldwide average Cu concentration in soils is 14 mg kg^−1^, while in Europe the average concentration is 12 mg kg^−1^ [126]. Copper is mobile in soils and its absorption is directly related to its concentration in the soil solution [44]. Plants can absorb Cu in huge amounts by roots and in minor amounts by shoots and leaves [127]. Mechanisms involved in Cu uptake are supposedly similar to those of Fe. Copper chelate reductases are encoded by ferric reductase oxidases 4 and 5 and Cu reduction occurs at the roots (Strategy I plants) where Cu is absorbed and transported by proteins of the COPT family. Copper uptake from soil depends almost exclusively on the protein COPT1, while COPT2 could act in the processes of Cu and Fe homeostasis and phosphate metabolism [27,128]. Plants can also absorb Cu through leaves, as observed by Stepien and Wojtkowiak [129] that after treating wheat plants with a foliar fertilization of copper sulphate in the amount of 0.2 kg Cu ha^−1^ (1% CuSO_4_ solution) obtained a 13% increase in the Cu content. On the other hand, the redox-active transition characteristic of Cu that makes it essential also contributes to its toxicity, since the reduction between Cu^2+^ and Cu^+^ catalyzes the production of toxic hydroxyl radicals that can damage DNA, cell membranes, and other biomolecules. Besides, damage to cell membranes can be reflected in low uptake of ions and water, so Cu toxicity can be indirectly expressed as growth inhibition and chlorosis caused by the generalized deficiency of nutrients and water [130]. Normally, crop species can tolerate a maximum of 20–30 mg kg^−1^ DW of Cu in leaves, but Cu-tolerant species can accumulate as much as 1000 mg kg^−1^ DW of Cu in leaves [44]. Moreover, foliar fertilization of Cu in maize should not exceed 100 g ha^−1^, since at higher doses, between 200 and 600 g ha^−1^, Barbosa et al. [131] noticed phytotoxic effects that caused growth and yield reduction up to 19% and 75%, respectively. In agriculture, Cu has been used for plant disease control for decades, a number of Cu formulations have been used as biocides to contain pathogens such as bacteria, fungi and in some cases, even invertebrates. In high concentrations, Cu interacts with nucleic acids, disrupting cell membranes of pathogens. In addition, direct application of Cu is used for seed treatment, to prevent seedling infections [132]. As shown in Table 1, among the few experiences in the biofortification of copper, Obrador et al. [133] conducted a study with spinach (*Spinacia oleracea* L.), var. ‘Viroflay Esmeralda’ applying eight different liquid fertilizers to the soil surface, with the irrigation water in a concentration ranging from 0 to 3 mg Cu kg^−1^ soil. Total Cu concentration in the dry matter of shoots increased by up to 450%, from 9.55 mg kg^−1^ (control treatment) to 52.51 mg kg^−1^ in the treatment where plants were submitted to 3 mg Cu kg^−1^ soil (as Cu-EDTA), a 4.54-fold increase (Table 1). However, at this dose they also noticed a 10% decrease in the dry matter yield. Instead, the dose 1 mg Cu kg^−1^ soil resulted in an increase in Cu content of 153% allowing also to obtain a yield increase of 71% when compared to the control. Regarding the chemical form, their results showed that the best fertilizers to increase Cu content in the edible part of spinach are Cu-DHE (Cu-diethylenetri-aminepentaacetate-N−2-hydroxyethyl-ethylenediamine-triacetate-ethylenediamine-tetraacetate) and especially Cu-EDTA. Curiously, in this study, even though the total concentration of Cu in spinach shoots was higher than the maximum concentration usually tolerated by plants, no visual phytotoxic symptoms and significant yield reductions were observed. In conclusion, Cu biofortification proved to be effective using different chelate forms and its potential as a biocide could benefit biofortification programs. In addition, when Cu biofortification is concerned attention must be made to the release of Cu in the soil substrate in relation to crop rotations and soil biological properties.

### 4.8. Silicon

Accumulating evidence from the last 30 years strongly suggests that silicon (Si) plays an essential role in bone formation and maintenance, improving the bone matrix quality and facilitating its mineralization. Increased intake of Si has been associated with increased bone mineral density and decreased osteoporosis [134]. Average daily dietary intake of Si is 20–50 mg for European population, the RDA has not been stablished; however, safe upper levels for humans have been recommended with a maximum range of 700–1750 mg day^−1^ [135]. Silicon is considered not essential for plant nutrition, but its inclusion in fertilization programs has proved to increase the crop tolerance to biotic and abiotic stressors [136], crop yield [137], or improve the absorption of macro- and microelements [138]. Silicon concentration in soil can vary depending on the type of soil. For example, alkaline soils containing sodium carbonate usually present a higher Si content. On average, the concentration of Si in soil is between 0.09 and 23.4 mg kg^−1^ [139]. If compared with other minerals, Si metabolism is still poorly understood. It seems that two main mechanisms of Si absorption coexist in plants, i.e., active and passive, whose relative contributions depend upon both plant species and external Si concentration [140]. This would explain the strong differences in Si concentration reported within tissues of different plants species [141]. In any case, Si is taken up by the roots as monosilicic acid with the involvement of channels belonging to the aquaporins’ group, so the water flow resulting from leaf transpiration seems to play a determinant role in defining the rate of Si absorption and transport within the plant [142]. Once absorbed, monosilicic acid is subsequently translocated to the shoot through the xylem flow, where Si is concentrated thanks to transpiration and polymerized to silica (SiO_2_), then deposited in the different tissues [143]. It has been reported in the Poaceae leaves that Si can be deposited both in mesophyll and epidermal cells, suggesting the coexistence of negative (transpiration-driven) and positive (though specific carriers) mechanisms controlling the Si accumulation process [144]. Plants markedly differ in their ability to accumulate Si in their various organs; concentrations ranging between 5 and 50 g kg^−1^ DW have been reported as critical for some species. The species with low mobilization capacity accumulate it in the roots and stems, while the species with high mobilization capacity accumulate Si in stems, leaves, fruits, and seeds [142]. Gao et al. [145] noticed that excessive Si supply (>2 mM) caused the formation of Si polymers on root surfaces, a feature that could affect nutrients uptake. In spite of the scarcity of available information, this aspect would deserve extensive study with reference to vegetable crops, due to their potential role as Si source in the human diet. Indeed, thanks to their usually low silicification capacity, vegetable crops are expected to contain high amounts of soluble Si, which is theoretically more available to be assimilated after ingestion, so potentially being optimal candidates as Si source in the human diet [142]. As shown in Table 1, as regards the leafy vegetables, in a study concerning six crops grown in a greenhouse floating system, namely *Brassica rapa* L. (tatsoi and mizuna group), *Ocimum basilicum* L., *Portulaca oleracea* L., *Cichorium intybus* L. and *Beta vulgaris* L. ssp. *vulgaris*, D’Imperio et al. [146] found an increased Si content in plant tissues by providing them up to 100 mg L^−1^ Si (as potassium metasilicate) in the nutrient solution, with basil reaching the highest content of Si (293 mg kg^−1^ FW, expressed as SiO_2_). Moreover, the authors found that Si became bioaccessible in all the considered species, in a range from 23% (basil) to 64% (chicory). In a different experiment concerning two leafy vegetables, namely chard (*Beta vulgaris* L. var. *cicla*) and kale (*Brassica oleracea* L. var. *acephala*) grown in a hydroponic system, De Souza et al. [147] compared the effects of two Si sources, namely potassium silicate and stabilized sodium potassium silicate with sorbitol, and four Si concentration in a foliar spray solution (from 0.00 to 2.52 g L^−1^). They found that in both species, the Si concentration in leaves linearly increased in response to Si concentration in the foliar spray solution, with the best biofortification results obtained by spraying potassium silicate. In a study concerning the green bean (*Phaseolus vulgaris* L.) cultivated in a hydroponic system, Montesano et al. [148] found that biofortified pods (obtained by adding 3.6 mM of Si as potassium metasilicate to a standard nutrient solution) showed a 310% increase of Si (from 853.8 to 2496.3 mg kg^−1^ DW) when compared to unbiofortified ones. Moreover, they found that the bioaccessibility of Si in biofortified pods was higher than control pods (25.1% vs. 7.6%), even after cooking them by steaming or boiling. The Si biofortification protocol of strawberry fruits (*Fragaria* × *ananassa* Duchesne ex Rozier) was studied by Valentinuzzi et al. [149], who cultivated for 16 weeks in a hydroponic system provided with a standard nutrient solution, or with nutrient solutions enriched with 50 or 100 mg L^−1^ of Si (as Na_2_SiO_3_). The authors found that providing 100 mg L^−1^ of Si allowed to maximize the metalloid concentration in strawberry fruits (which increased from 6.44 up to 85 g kg^−1^ DW) without compromising crop yield. However, the they observed a decrease in total phenols and an increase in the content of flavonols in response to the highest Si supply. Overall, biofortification with Si using K silicate proved to effectively increase the mineral content in vegetables. In addition, its possible role as plant protector and its ability to improve the mineral status of the plant, both make Si a key element in biofortification programs.

## 5. Discussion and Future Trends

**Figure 1:**
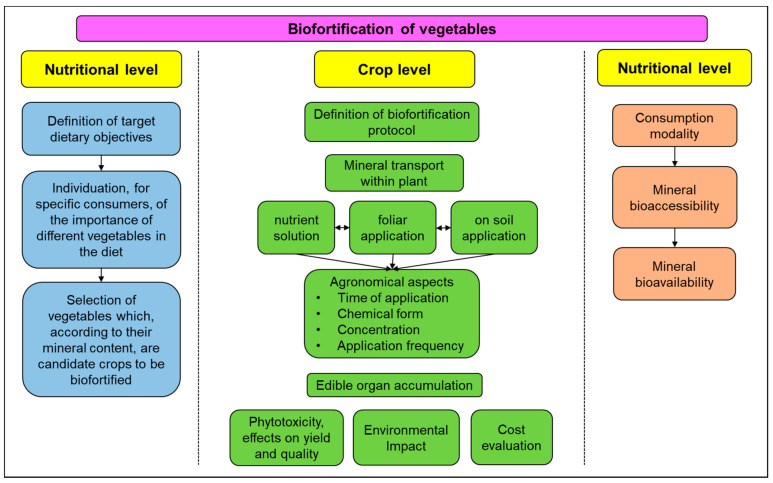
Key aspects to be considered in the agronomic mineral biofortification.

The evidence discussed above pointed out that biofortification should be contemplated as a promising strategy to face malnutrition in many circumstances. Biofortification can help to obtain products designed according to the needs of two categories of target consumers (Figure 1). The first concerns products enriched with minerals that can fulfil specific functional needs; this is the case of vegetables richer in one or more minerals to counter the deficiencies related to ordinary diet or new consumer habits. (e.g., vegans). Besides vitamins, in fact, vegan diets feature an inadequate content of calcium, potassium, iron, iodine, and magnesium [150]. A second target concerns products with premium quality or superfood aimed at improving health as a whole. This would satisfy the need of an increasing group of health-conscious consumers who look at plant-based foods, especially vegetables, as a sort of medicine to prevent the insurgence of chronic diseases.

Agronomic biofortification is comparatively simpler than other methods and potentially suitable for immediate results. However, the available studies on agronomic fortification of vegetables are of a considerable number only for few crops (e.g., lettuce, tomato, spinach, and *Brassica* spp.) and for few mineral elements (e.g., selenium, iodine). For these elements, aspects related to the form, application modality, concentration, and timing have been clarified for most important crops. For all the considered elements, and particularly for selenium and iodine, the biofortification adopting soilless crops or on soil fertigated crops have been mostly considered. In some cases the model describing the accumulation in relation to the application has also been described [151]. For some other mineral elements considered in this review, important as well in human nutrition (e.g., Fe), information is still lacking.

On the other hand, even when empirical evidence on biofortification showed a significant increase in the concentration of the mineral elements, the fortification is not economically worthwhile. In addition, an effective biofortification protocol is based on regular and frequent applications and a negative environmental impact cannot be excluded [32]. Besides, the step between biofortification and plant toxicity effects can be narrow and applications targeting the accumulation of essential micronutrients must be adjusted to avoid negative effects on plant growth [38].

The application of biofortifying elements poses some problems related to the interaction with other factors at soil level (e.g., phytoavailability) and at plant level (e.g., competition with other elements) [152]. In many studies the traditional fertigation approach is adopted, rather than foliar spray, which can be more cost effective and environmentally friendly. Indeed, foliar fertilization represents the simplest and fastest method for the application of mineral elements used for the biofortification of vegetables; but, the effectiveness depends on the used plant organ and the mobility of the element inside the plant. To face some of these problems, technical innovations such as precision agriculture, soilless cultivation, etc., may help in defining more efficient biofortification protocols.

There are only few biofortified vegetable products already present on the market (e.g., selenium enriched potato, carrot and onion, ‘Selenella’ from Consorzio Patata Italiana di Qualità Soc. Cons., IT, iodine biofortified potato, ‘Iodì’ from the Pizzoli group, IT, selenium enriched brussels sprouts from Marks and Spencer, UK, etc.). It is clear that mostly iodine and selenium have been commercially considered as biofortification elements, probably because a more efficient accumulation system and for their lower toxicity at plant level. In the future, besides a broad choice of diversified vegetables, it is expected that the market will have biofortified products richer in more than one mineral. Therefore, research that comprises simultaneous biofortification is essential. In addition, further elements are being studied and are expected to be object of biofortification in the future (e.g., lithium, vanadium, etc.). In this regard, biofortification using Li-sulfate and Li-hydroxide was effective in increasing Li content in lettuce plants [153].

Based on the results in literature, biofortification is not expected to fully control mineral element deficiencies or eradicate them, but it complements other interventions to provide micronutrients to people. To be effective, a biofortification program should be based on very appropriate planning concerning: health and nutrition investigation, nutritional habits, design and validation of sustainable biofortification protocols, estimation of positive effect on health. Concerning biofortification protocols, the attention should be paid to those crops having an element content high enough to be conveniently targeted, and that prove to significantly benefit from mineral elements application.

In the reviewed literature most attention has been posed on the content of specific elements in plant edible portion but key concepts like bioaccessibility and bioavailability were seldomly considered. The first regards the nutrient fraction released from the food and available for absorption by the intestinal cells, while the latter expresses the amount of nutrients actually absorbed and therefore available for utilization in physiological functions [154,155]. While macronutrients (proteins, carbohydrates, and fats) are degraded and absorbed by specific and well-known biochemical mechanisms, phytochemicals and minerals are absorbable without biotransformation and often without a specific carrier [156,157]. The consequence of this poorly developed intestinal transport system is that the actual absorption of phytochemicals and minerals is deeply dependent on the food matrix. To modulate mineral bioavailability, attention should be devoted to those substances (e.g., vitamin C, β-carotene, oxalic acid, polyphenols, etc.) stimulating or inhibiting bioavailability [27,158]. Furthermore, some chemical bonds with other components in the food or the physical entrapping inside intact plant cell walls can dramatically decrease the bioaccessible and bioavailable fractions of phytochemicals and minerals [159].

## 6. Conclusions

In conclusion, the production of mineral-dense vegetables will deserve a prominent place in the coming years. Agronomic biofortification, even if it involves expensive experimental activities, represents the only strategy in the case of vegetables, for which genetic improvement programs would be rather complex and not very convenient due to the high rate of varietal turnover. The main challenges for agronomic biofortification in the immediate future will rely on the efficiency of fertilization process and bioavailability of minerals, the high cost of some specific chemical formulations, the possible yield losses due to biofortification-induced alterations of plant metabolism, and the potential environmental/health impact deriving from new agronomic protocols (as in the case, for example of copper and selenium). Deeper knowledge in these areas must be considered indispensable to achieve sound conclusions about the costs/benefits of biofortification.

The papers discussed in this review report promising results for several minerals and pillar vegetables in the human diet; however, the results are not entirely consistent and coherent. The future activities, beyond their specific scientific relevance, should be planned in a broader context, adopting an approach involving also farmers, traders, nutritionists, and educators. Evidence from research shows that farmers are willing to cultivate and commercialize biofortified crops and the few and selected products available in the market demonstrates that consumers are favorably eating them. Furthermore, nutrition specialists together with health educators can also have an impact on the population’s eating habits and contribute to increase the consumption of the target vegetables. Such an approach, thanks also to the nutritional importance of vegetables, will certainly have a significant impact on improving human diet.

## Figures and Tables

**Table 1:** Response of some vegetable crops to biofortification ^(1)^.

Element	Crop	Scopus^®^ Papers (n.)	Average Concentration ^(2)^ (mg kg^−1^ FW)		Average Increase	Application Dose to Roots or Leaves (mg L^−1^)	
			Min	Max		Min	Max
Ca	Basil	1	950	1100	0.2-fold	100	200
Ca	Endive	1	1020	1080	0.1-fold	100	200
Ca	Indian colza	2	928	3000	2.2-fold	6	1603
Ca	Lettuce	2	695	2683	2.9-fold	0	800
Ca	Mizuna	1	1250	1400	0.1-fold	100	200
Ca	Potato	1	144	245	0.7-fold	350	5200
Ca	Tatsoi	1	1100	1150	1.1-fold	100	200
Mg	Indian colza	2	290	1059	2.7-fold	4	486
Mg	Onion	1	652	1627	1.5-fold	0	150
I	Basil	2	1	287	>100-fold	0.1	127
I	Cabbage	3	0.1	2.5	34.4-fold	0.1	0.6
I	Carrot	7	0.1	7.8	>50-fold	1	50
I	Chinese cabbage	3	0.1	48.7	>100-fold	0.1	50
I	Cowpea	2	4	1566	>100-fold	0.7	15
I	Lettuce	18	2	42.0	17.9-fold	0.1	50
I	Mizuna	2	0	1.0	>50-fold	0.7	1.1
I	Mustard	2	0	0.4	41-fold	0.7	1.1
I	Onion	1	0	1.0	>50-fold	0	5
I	Potato	3	0.1	0.7	11.3-fold	0.6	5
I	Spinach	8	4.5	22.4	4.0-fold	1	5.1
I	Tomato	5	0.1	12.0	>100-fold	1	634
Zn	Arugula microgreens	1	3.0	70	22.3-fold	0	10
Zn	Broccoli	1	9.4	133	13.2-fold	121	408
Zn	Cabbage	4	4.1	39.1	8.6-fold	5	260
Zn	Carrot	1	42.1	802	18.1-fold	2.8	303
Zn	Indian colza	1	2.5	167	>50-fold	0	32.7
Zn	Kale	1	5.8	167	27.8-fold	2.8	303
Zn	Lettuce	3	2.2	30.4	12.8-fold	5.2	60
Zn	Okra	1	3.0	5.0	0.7-fold	2.8	303
Zn	Onion	1	2.5	7.8	2.1-fold	0	10
Zn	Potato	3	2.7	4.9	0.8-fold	9.7	250
Zn	Red cabbage microgreens	1	2.5	75	29-fold	0	10
Zn	Red mustard microgreens	1	2.1	92	42.8-fold	0	10
Se	Basil	4	0	8.3	>100-fold	2	12
Se	Broccoli	1	1.1	19.2	15.7-fold	10	100
Se	Carrot	3	0.1	1.8	35-fold	0.3	3.9
Se	Chard	1	0	0.5	45-fold	0	10
Se	Cucumber	1	0	0.2	7.6-fold	0	30
Se	Endive	1	0.1	1.2	23.6-fold	0.3	0.6
Se	Garlic	2	0.1	6.1	>50-fold	0.1	15
Se	Indian mustard	1	0	0.5	>50-fold	0	50
Se	Lettuce	12	0.1	6.9	>100-fold	0.5	20
Se	Onion	3	0.4	17.7	49.5-fold	2.0	20
Se	Potato	4	0.1	1.6	16.6-fold	0.5	0.8
Se	Radish	4	0.3	18.2	>50-fold	1	23.7
Se	Spinach	2	0.1	2.2	21.1-fold	0.2	0.3
Se	Strawberry	1	0.5	3.0	5.2-fold	0	4
Se	Tomato	3	0.3	3.4	9.1-fold	5	20
Se	Turnip	1	0.4	10.6	24.3-fold	0.2	2
Fe	Arugula microgreens	1	4.9	111	21.6-fold	0	40
Fe	Lettuce	1	2.3	4.3	0.9-fold	0.8	112
Fe	Red cabbage microgreens	1	7.7	448	>50-fold	0	40
Fe	Red mustard microgreens	1	4.9	323	>50-fold	0	40
Fe	Sweet potato	1	185	253	0.4-fold	0	100
Cu	Spinach	1	0.5	3.0	4.5-fold	0	3
Si	Basil	1	41.2	293	6.1-fold	0	100
Si	Chard	1	500	1450	1.9-fold	0	2.5
Si	Chicory	2	17.2	95	4.5-fold	0	101
Si	Green bean	1	57	252	3.4-fold	0	101
Si	Kale	1	700	2800	3-fold	0	2.5
Si	Mizuna	1	20	110	4.5-fold	0	100
Si	Purslane	1	14.8	98	5.6-fold	0	100
Si	Strawberry	1	475	8075	16-fold	0	100
Si	Swiss chard	1	18	145	7.1-fold	0	100
Si	Tatsoi	1	18	70	2.9-fold	0	100

^(1)^ The list reports the most representative horticultural crops. In this and in the following tables, data refer to research on Scopus**^®^** using “biofortification” and “vegetables” as keywords performed in November 2020. Papers which tested more than one species were counted more than one time. ^(^^2)^ Calculated in the edible portion.

**Table 2:** Chemical forms of each mineral used in the biofortification of some vegetable crops.

	Basil	*Brassica* spp.	Carrot	Lettuce	Onion	Potato	Radish	Spinach	Tomato
Ca	Calcium phosphate, calcium chloride	Calcium chloride		Calcium chloride		Calcium chloride, calcium nitrate			
Cu								Cu-EDTA ^1^	
Fe		Iron sulphate		Fe-EDDHA ^2^					
I	Potassium iodide, potassium iodate	Potassium iodide, potassium iodate	Potassium iodide, potassium iodate	Potassium iodide, potassium iodate	Potassiumiodide	Potassium iodide, potassium iodate		Potassium iodide, potassium iodate	Potassium iodide, potassium iodate
Mg		Magnesium chloride, magnesium sulphate			Magnesium sulphate				
Se	Sodium selenate	Sodium selenate, sodium selenite	Sodium selenate, sodium selenite	Sodium selenate, sodium selenite	Sodium selenate	Sodium selenate, sodium selenite	Sodium selenate, sodium selenite	Sodium selenate, sodium selenite	Sodium selenate, sodium selenite
Si	Potassium meta silicate	Potassium silicate, sodium silicate							
Zn		Zinc nitrate, Zinc sulphate	Zinc oxide, zinc sulphate, Zinc EDTA ^1^	Zinc sulphate	Zn-AML ^3^, Zn-EDDHSA ^4^, Zn-EDTA ^1^, Zn-PHP ^5^, Zn-HEDTA ^6^, Zn-EDTA ^1^-HEDTA ^6^, Zn-DTPA ^7^-HEDTA ^6^-EDTA^1^, Zn-EDDS ^8^	Zinc nitrate, zinc oxide, zinc sulphate, Zn-EDTA ^1^			

^1^ Ethylenediaminetetraacetic acid, ^2^ Ethylenediamine-N,N′-bis(2-hydroxyphenylacetic acid), ^3^ Aminolignosulfonate, ^4^ ethylenediamine-di-(2-hydroxy-5-sulfophenylacetate), ^5^ Polyhydroxyphenylcarboxylate, ^6^ N-2-hydroxyethyl-ethylenediaminetriacetate, ^7^ Diethylenetriaminepentaacetate, ^8^ Ethylenediamine disuccinate. The list includes those papers that reported the initial and final concentration of mineral element in the edible part of vegetables.

## Data Availability

Data sharing not applicable.
